# Supplementary material for: The relative contribution of close-proximity contacts, shared classroom exposure and indoor air quality to respiratory virus transmission in schools
Source: Nat Commun. 2025 Nov 27;16:11678. doi: 10.1038/s41467-025-66719-3 (PMC12749238; doi:10.1038/s41467-025-66719-3)
Supplement: Supplementary file 3 — Reporting Summary [file 41467_2025_66719_MOESM3_ESM.pdf]

nature portfolio

Corresponding author(s): Lukas Fenner

Last updated by author(s): 9/23/2025

Reporting Summary

Nature Portfolio wishes to improve the reproducibility of the work that we publish. This form provides structure for consistency and transparency in reporting. For further information on Nature Portfolio policies, see our Editorial Policies and the Editorial Policy Checklist.

Statistics

For all statistical analyses, confirm that the following items are present in the figure legend, table legend, main text, or Methods section.

n/a	Confirmed	
☐	☒	The exact sample size (*n*) for each experimental group/condition, given as a discrete number and unit of measurement
☐	☒	A statement on whether measurements were taken from distinct samples or whether the same sample was measured repeatedly
☐	☒	The statistical test(s) used AND whether they are one- or two-sided<br>*Only common tests should be described solely by name; describe more complex techniques in the Methods section.*
☐	☒	A description of all covariates tested
☐	☒	A description of any assumptions or corrections, such as tests of normality and adjustment for multiple comparisons
☐	☒	A full description of the statistical parameters including central tendency (e.g. means) or other basic estimates (e.g. regression coefficient) AND variation (e.g. standard deviation) or associated estimates of uncertainty (e.g. confidence intervals)
☒	☐	For null hypothesis testing, the test statistic (e.g. *F*, *t*, *r*) with confidence intervals, effect sizes, degrees of freedom and *P* value noted<br>*Give P values as exact values whenever suitable.*
☒	☐	For Bayesian analysis, information on the choice of priors and Markov chain Monte Carlo settings
☒	☐	For hierarchical and complex designs, identification of the appropriate level for tests and full reporting of outcomes
☒	☐	Estimates of effect sizes (e.g. Cohen's *d*, Pearson's *r*), indicating how they were calculated

Our web collection on statistics for biologists contains articles on many of the points above.

Software and code

Policy information about availability of computer code

Data collection	Epidemiological and environmental data were entered electronically on the secure web platform REDCap (https://project-redcap.org/).
Data analysis	Pairwise accelerated failure time models were estimated using the TranStat package version 0.3.7 (60). All analyses were performed in R version 4.2.

For manuscripts utilizing custom algorithms or software that are central to the research but not yet described in published literature, software must be made available to editors and reviewers. We strongly encourage code deposition in a community repository (e.g. GitHub). See the Nature Portfolio guidelines for submitting code & software for further information.

Data

Policy information about availability of data

All manuscripts must include a data availability statement. This statement should provide the following information, where applicable:

- Accession codes, unique identifiers, or web links for publicly available datasets
- A description of any restrictions on data availability
- For clinical datasets or third party data, please ensure that the statement adheres to our policy

De-identified close-proximity data and all genomic sequences generated from the data collected during this study are available at osf.io/naut4. Sequences used in the main analysis detailed in this manuscript are further available on GenBank under accession numbers detailed in Supplementary Text C. Genomic viral sequences collected as part of the Swiss Respiratory Virus Sequencing study are available in BioProject PRJEB83635 (https://www.ncbi.nlm.nih.gov/bioproject/PRJEB83635)

under the accession numbers detailed in Supplementary Text C and Tables S3-S4. Global genomic viral sequences are available from GenBank (https://www.ncbi.nlm.nih.gov/genbank/) under the accession numbers detailed in Supplementary Text C and Tables S5-S6. Restrictions on the availability of other personal data apply but are necessary to maintain the confidentiality of participants. The data can be made available upon request, subject to approval by the local ethics committee and the Technology Transfer Office of the University of Bern. Requests will be given expedited review, but the timeframe depends on the review time of the involved parties (contact: University of Bern, info.ispm@unibe.ch).

Research involving human participants, their data, or biological material

Policy information about studies with human participants or human data. See also policy information about sex, gender (identity/presentation), and sexual orientation and race, ethnicity and racism.

Reporting on sex and gender	Descriptive results about viral infections and school absences were reported separately by sex in Table 1 of the manuscript.
Reporting on race, ethnicity, or other socially relevant groupings	No data collected
Population characteristics	Study participants were aged 13 to 15 years but individual age was not collected. Genomic sequencs were collected for positive saliva tests linked to individual study participants. All individual-level data was anonymised and restrictions on the availability of the data apply (see Data availability statement above).
Recruitment	From the participating school, all students willing to participate in the collection of molecular, epidemiological, and proximity data were included, and written informed consent was obtained from the students and their caregivers.
Ethics oversight	The study was approved by the Ethics Committee of the Canton of Bern, Switzerland (reference no. 2023-02035).

Note that full information on the approval of the study protocol must also be provided in the manuscript.

Field-specific reporting

Please select the one below that is the best fit for your research. If you are not sure, read the appropriate sections before making your selection.

☐ Life sciences ☐ Behavioural & social sciences ☒ Ecological, evolutionary & environmental sciences

For a reference copy of the document with all sections, see nature.com/documents/nr-reporting-summary-flat.pdf

Ecological, evolutionary & environmental sciences study design

All studies must disclose on these points even when the disclosure is negative.

Study description	We conducted a six-week longitudinal study during the peak of the respiratory virus season in winter of 2023/24 in a Swiss school. The study comprised four classes from the same grade level, all located on the same building floor to allow for interactions within and between classes. We used a comprehensive probabilistic framework combining molecular data, epidemiological data and assumptions, and genetic proximity of respiratory viruses to reconstruct plausible transmissions between students (Figure 1). Furthermore, we detected close-proximity interactions using wearable sensors, monitored indoor air quality with aerosol devices and CO2 monitors, and considered social factors possibly associated with transmission outside school. Using pairwise accelerated failure time models, we estimated the association of respiratory virus transmission with time in close-range proximity, time spent in shared classrooms, time with suboptimal air quality (CO2 levels or particulate matter mass concentrations above recommended levels), and social factors (household siblings and extracurricular activities). We evaluated the relative contribution of each risk factor both overall and within individual classes, identifying key drivers of respiratory virus transmission in schools.
Research sample	Four classes (67 students aged 14–15 years) of a secondary school in the canton of Bern, Switzerland.
Sampling strategy	All students willing to participate in the collection of molecular, epidemiological, and proximity data were included, and written informed consent was obtained from the students and their caregivers. A sample size calculation for ethics approval was done before the study as follows. Dividing transmission events into a prolonged-close contact and a long-range transmission group, then 154, 25, or 10 events would be needed to estimate a small, medium, or large effect, respectively, assuming a significance level of 5% and statistical power of 80%. Considering a common class size of 20 students of which 50% will experience a transmission event over the study period, we can expect 20 or 30 transmission events with two or three participating classes. Thus, with four participating classes the study was sufficiently powered to detect a medium to large effect of prolonged-close contact on the risk of transmission (primary outcome).
Data collection	We detected close-proximity interactions using wearable sensors and monitored indoor air quality with aerosol devices and CO2 monitors. Saliva samples were collected three times per week and school absences daily both by our local study team. Social factors were collected via a survey both at the start and end of the study.
Timing and spatial scale	Data collected during the main season for respiratory virus infections in winter 2024/2025 for six weeks from January 22 to March 8, 2024. Data was collected in four classes that were on the same building floor of secondary school, which was located in an urban environment in the canton of Bern, Switzerland.

Data exclusions	No exclusions
Reproducibility	Not applicable
Randomization	Not applicable
Blinding	Neither the students nor the study team were aware of the detected virus infections during the study.

Did the study involve field work? ☐ Yes ☐ No

Field work, collection and transport

Field conditions	Data collected in four classrooms during winter, with normal room temperature of around 20 degrees Celsius. Repeated saliva sampling was done three times per week using saline saliva collection kits under the guidance of our study team. Samples were transported to the clinical microbiology laboratory and stored at −80 °C until further processing.
Location	The school was located in an urban environment in the Canton of Bern, Switzerland.
Access & import/export	Not applicable
Disturbance	Not applicable

Reporting for specific materials, systems and methods

We require information from authors about some types of materials, experimental systems and methods used in many studies. Here, indicate whether each material, system or method listed is relevant to your study. If you are not sure if a list item applies to your research, read the appropriate section before selecting a response.

Materials & experimental systems

n/a	Involved in the study	
☒	☐	Antibodies
☒	☐	Eukaryotic cell lines
☒	☐	Palaeontology and archaeology
☒	☐	Animals and other organisms
☒	☐	Clinical data
☒	☐	Dual use research of concern
☒	☐	Plants

Methods

n/a	Involved in the study	
☒	☐	ChIP-seq
☒	☐	Flow cytometry
☒	☐	MRI-based neuroimaging

Plants

Seed stocks	*Report on the source of all seed stocks or other plant material used. If applicable, state the seed stock centre and catalogue number. If plant specimens were collected from the field, describe the collection location, date and sampling procedures.*
Novel plant genotypes	*Describe the methods by which all novel plant genotypes were produced. This includes those generated by transgenic approaches, gene editing, chemical/radiation-based mutagenesis and hybridization. For transgenic lines, describe the transformation method, the number of independent lines analyzed and the generation upon which experiments were performed. For gene-edited lines, describe the editor used, the endogenous sequence targeted for editing, the targeting guide RNA sequence (if applicable) and how the editor was applied.*
Authentication	*Describe any authentication procedures for each seed stock used or novel genotype generated. Describe any experiments used to assess the effect of a mutation and, where applicable, how potential secondary effects (e.g. second site T-DNA insertions, mosiacism, off-target gene editing) were examined.*
